# Dynamic brainstem and somatosensory cortical excitability during migraine cycles

**DOI:** 10.1186/s10194-022-01392-1

**Published:** 2022-02-05

**Authors:** Fu-Jung Hsiao, Wei-Ta Chen, Li-Ling Hope Pan, Hung-Yu Liu, Yen-Feng Wang, Shih-Pin Chen, Kuan-Lin Lai, Gianluca Coppola, Shuu-Jiun Wang

**Affiliations:** 1 Brain Research Center, National Yang Ming Chiao Tung University, Taipei, Taiwan; 2 School of Medicine, National Yang Ming Chiao Tung University, Taipei, Taiwan; 3 Department of Neurology, Neurological Institute, Taipei Veterans General Hospital, 201, Shihpai Rd Sec 2, Taipei, 112 Taiwan; 4 Department of Neurology, Keelung Hospital, Ministry of Health and Welfare, Keelung, Taiwan; 5 Department of Medico-Surgical Sciences and Biotechnologies, Sapienza University of Rome Polo Pontino, Latina, Italy

**Keywords:** Brainstem, Primary somatosensory cortex (S1), Migraine cycle, Excitability, Inhibition

## Abstract

**Abstract:**

**Background:**

Migraine has complex pathophysiological characteristics and episodic attacks. To decipher the cyclic neurophysiological features of migraine attacks, in this study, we compared neuronal excitability in the brainstem and primary somatosensory (S1) region between migraine phases for 30 consecutive days in two patients with episodic migraine.

**Methods:**

Both patients underwent EEG recording of event-related potentials with the somatosensory and paired-pulse paradigms for 30 consecutive days. The migraine cycle was divided into the following phases: 24–48 h before headache onset (*Pre2*), within 24 h before headache onset (*Pre1*), during the migraine attack (*Ictal*), within 24 h after headache offset (*Post1*), and the interval of ˃48 h between the last and next headache phase (*Interictal*). The normalised current intensity in the brainstem and S1 and gating ratio in the S1 were recorded and examined.

**Results:**

Six migraine cycles (three for each patient) were analysed. In both patients, the somatosensory excitability in the brainstem (peaking at 12–14 ms after stimulation) and S1 (peaking at 18–19 ms after stimulation) peaked in the *Pre1* phase. The S1 inhibitory capability was higher in the *Ictal* phase than in the *Pre1* phase.

**Conclusion:**

This study demonstrates that migraine is a cyclic excitatory disorder and that the neural substrates involved include the somatosensory system, starting in the brainstem and spanning subsequently to the S1 before the migraine occurs. Further investigations with larger sample sizes are warranted.

## Introduction

Migraine is the most prevalent neurological disorder, and it is the second most debilitating disease worldwide [1]. It has complex pathophysiological characteristics and episodic attacks of moderate to severe headache accompanied by nausea, vomiting, or hypersensitivity to sensory stimuli such as light, sound, and odour. Mounting evidence suggests that migraine symptoms are determined by complex interactions among genetic, environmental, hormonal, and other endogenous factors [2, 3]. Although the underlying neuropathological mechanism remains unclear, a fundamental imbalance of neuronal excitability (i.e. neuronal dysexcitability) may play a pivotal role [4]. In brain excitability studies, functional changes during migraine attacks have included hyperresponsivity to repeated sensory stimuli, altered recruitment of neuronal networks, and impaired habituation [4–9]. However, research results on excitability have differed because of diverse intervals from migraine attacks among study populations [9, 10]. To resolve this challenge, brain excitability dynamics should be recorded throughout entire migraine cycles.

In pioneering migraine studies, Schulte and colleagues used 30-day functional MRI (fMRI) after trigeminal nociceptive stimulation or in the resting state to investigate prodromal functional changes in neural activity and functional coupling within the brainstem, hypothalamus, nucleus accumbens, amygdala, hippocampus, and visual cortex [11–13]. Hypothalamus–brainstem connectivity has been implicated as a driver of migraine attacks [11], and enhanced brainstem activation may be a marker of a migraine attack (i.e. cyclic activation) [11–13]. These findings implicate the dysfunction of the brainstem and its relevant network in migraine, suggesting that such neurological changes led to this complex brain disorder [14]. However, fMRI does not directly measure neural activity [15, 16], and blood oxygen level–dependent signals are limited by low temporal resolution. Additionally, continuous trigeminal nociceptive stimulation could trigger adverse plastic adaptive changes in several brain areas, which may have biased the results [17]. Therefore, somatosensory-evoked potentials (SSEPs) may be more appropriate for examining brain excitability dynamics during the entirety of a migraine cycle because they exhibit superior temporal resolution, can be recorded noninvasively and with innocuous stimulation, and are direct reflection of neural activity [4]. Moreover, impaired brainstem function even in the interictal period was noted in one EEG migraine study [18].

Central sensitisation of the primary somatosensory cortex (S1) and impaired response habituation to repetitive afferent stimuli are also crucial neurophysiological occurrences in migraine [4, 19]. In our recent studies [6, 20], S1 gating, a habituation-related but more basic protective mechanism against brain sensory overload, was altered in patients with migraine, and interictal inhibitory function (i.e. gating ratio) was linked to migraine severity. However, to determine the cyclic characteristics of migraines, S1 inhibition throughout the migraine cycle should be analysed longitudinally.

As mentioned, neuronal excitability and inhibition are neurophysiological indicators in migraines, especially in the brainstem and S1. To determine the cyclic neurophysiological features of migraines, this study examined the evoked activity of the brainstem and S1 region in various migraine phases; the evoked activity was recorded each morning for 30 consecutive days in patients with episodic migraine (EM) by using EEG techniques with nonpainful somatosensory stimulation.

## Methods

### Participants

Two female patients (aged 26 and 32 years) who were diagnosed with EM without aura according to the third edition of the *International Classification of Headache Disorders* [21], were recruited from the Headache Clinic of Taipei Veterans General Hospital. Neither patient had a history of systemic or major neurological disease. Both patients had normal results on physical and neurological examinations, and both were right handed. Both willingly joined this study for a 30-day period and provided signed informed consent. Both completed a daily headache diary and EEG recordings. Both were asked to refrain from acute medication consumption for at least 16 h before the EEG recordings. They did not take any preventive medication, neither during the studying period nor before the study. The hospital’s institutional review board approved the study protocol (VGHTPE: IRB 2019–07-001B).

### Study design

During the 30-day period, headache status (headache or no headache), headache characteristics (pain rating and pain location), menstrual cycle status, use of analgesic medications, and presence of premonitory and other migraine-associated symptoms were assessed daily. The daily levels of pain intensity, anxiety, depression, and stress were subjectively rated on a visual analogue scale anchored at 0 and 10. Moreover, cutaneous allodynia was evaluated before the study using a 17-item questionnaire as reported in our related work [22]. Additionally, each participant underwent EEG recording of event-related potentials with standardised electrical somatosensory stimulation between 10:00 a.m. and 12:00 p.m. each day for 30 days. The somatosensory and paired-pulse paradigms were employed. After the 30 days of recordings, pain-free T1 structural images were acquired for further EEG source analysis.

For a migraine cycle to be analysed, it must feature a headache attack preceded by at least 3 pain-free days, not coincide with another migraine cycle (to exclude possible postdromal effects), and have acceptable EEG data recorded without any technical problems. Thus, the number of analysed attacks could differ from the number of observed attacks. We divided the peri-ictal, ictal, and interictal periods into phases according to their relative time as follows [12, 21]: 24–48 h before headache onset was *Pre2*, within 24 h before headache onset was *Pre1*, during the migraine attack was *Ictal*, within 24 h after headache offset was *Post1*, and the period ˃48 h from the last and next headache phase was *Interictal*. Any day with a tension-type headache (TTH) attack during the interictal phase was excluded from analysis.

### EEG recording and analysis

Scalp EEG data were collected from an EEG cap housing a 64-electrode BrainVision actiCAP system (Brain Products GmbH, Munich, Germany) that covered the entire brain according to the extended International 10–20 system [23]. Active circuits for impedance conversion were integrated into the slim actiCAP electrodes, enabling high signal quality at higher impedances than conventional passive electrodes allow. The electrodes were referenced online to an electrode placed on the Fz plane, and a common ground connection was established at the FPz site. The EEG signals were amplified and digitised using a BrainAmp DC amplifier (Brain Products GmbH, Munich, Germany) linked to Brain Vision Recorder software (version 2.1, Brain Products GmbH, Munich, Germany).

Somatosensory-evoked and paired-pulse stimulation was delivered to each patient during the daily EEG recordings. For the two stimulation tests, a Digitimer DS7A device (Digitimer, Welwyn Garden City, Hertfordshire, UK) with constant-current square-wave pulses (0.2-ms width, proximal cathode) was used; the intensity of electrical stimulation of the right median nerve at the wrist was twice the subjective sensory threshold, and no pain response or visible twitching of the flexor digitorum superficialis was elicited. The patients were comfortably seated on a chair in an illuminated room and asked to remain awake with their eyes closed. Evoked brain activity was continually recorded at a digital sampling rate of 1000 Hz. For the somatosensory test, electrical stimulation was delivered at 4 Hz/s to collect 1000 samples of SSEPs, including a prestimulus baseline of 50 ms and poststimulus measurement of 100 ms, for a sufficient number of samples to reliably determine average brainstem responses [24]. For the paired-pulse paradigm, the stimulation comprised paired pulses applied to the right median nerve with an interstimulus interval of 500 ms and an interpair interval of 8 s [25]. The length of each recorded trial, except for the prestimulus baseline of 50 ms, was 150 ms. At least 100 artefact-free responses to the first and second pulses of the paired stimuli (hereafter referenced as ‘first response’ and ‘second response’, respectively) were recorded. Notably, to avoid fatigue, a 10-min break was granted between the two tests.

Distributed current source modelling of EEG data was performed using depth-weighted minimum norm estimates (MNEs) [6, 20, 26], which accurately resolve source localisation, even for deep generators [27, 28]. The neuronal dynamics of cortical and subcortical sources were determined using a deep brain model that describes the signal patterns generated by a unit dipole, realistically distributing current dipoles over the neocortex and subcortical structures [28]. This forward model uses the symmetric boundary element method [29], which provides more accurate results than spherical models provide. Structural brain imaging was performed using a 3 T MR system (Magnetom Tim Trio; Siemens, Malvern, PA, USA) with the following parameters: repetition time, 9.4 ms; echo time, 4 ms; recording matrix, 256 × 256 pixels; field of view, 256 mm; and slice thickness, 1 mm. The shapes of surfaces separating the scalp, skull, and brain compartments were identified using FreeSurfer 7.0 software (Harvard, Cambridge, MA, USA), which was also used for the subcortical segmentation of brain volume. The inverse operator of MNE analysis was used to estimate the distribution of the current sources that account for the data recorded at the electrodes. The aforementioned analysis resulted in distributed and dynamic brain activation that could be mapped onto the reconstructed surface and volume for each patient; consequently, the time-varying current intensity could be extracted from the brainstem (‘volume scout’ function) and S1 (‘surface scout’ function). In the somatosensory-evoked task, the peak current intensity in the brainstem at 12–13 ms and S1 at 17–18 ms was obtained [18]. To highlight the components from the subcortical generators, the current density values were transformed into *z* scores which represented the number of standard deviations from the baseline level. To compare the dynamic neural excitability of different migraine attacks, the current density in each phase of the migraine cycle (*Pre2*, *Pre1*, *Ictal*, *Post1*, and *Interictal* phases) was also normalised in relation to the ictal day (*Ictal*) of the cycle. For the paired-pulse task, the peak S1 current density was obtained, and the gating ratio was subsequently determined (current density of second response/current density of first response) [6, 20, 25]. Data analysis was performed using Brainstorm software [30], which has been partially described in our previous papers [6, 20].

### Statistical analysis

The normalised current intensity and gating ratio were obtained from the two patients’ selected migraine cycles and examined by phase (*Pre2*, *Pre1*, *Ictal*, *Post1*, and *Interictal*). Nonparametric tests were applied because the data were not normally distributed. Specifically, the Kruskal–Wallis test was initially used to compare phases. Second, the Wilcoxon signed-rank test was used to compare the normalised current intensity and gating ratio of the *Pre2* and *Pre1*, *Pre1* and *Ictal*, and *Ictal* and *Post1* phases. Finally, the Mann–Whitney test was used to compare the *Interictal* phase with the other phases within the cycle. All tests were two tailed, and the significance level was *P* < 0.05. Bonferroni correction was applied for multiple comparisons.

## Results

### Symptom description

**Fig. 1:**
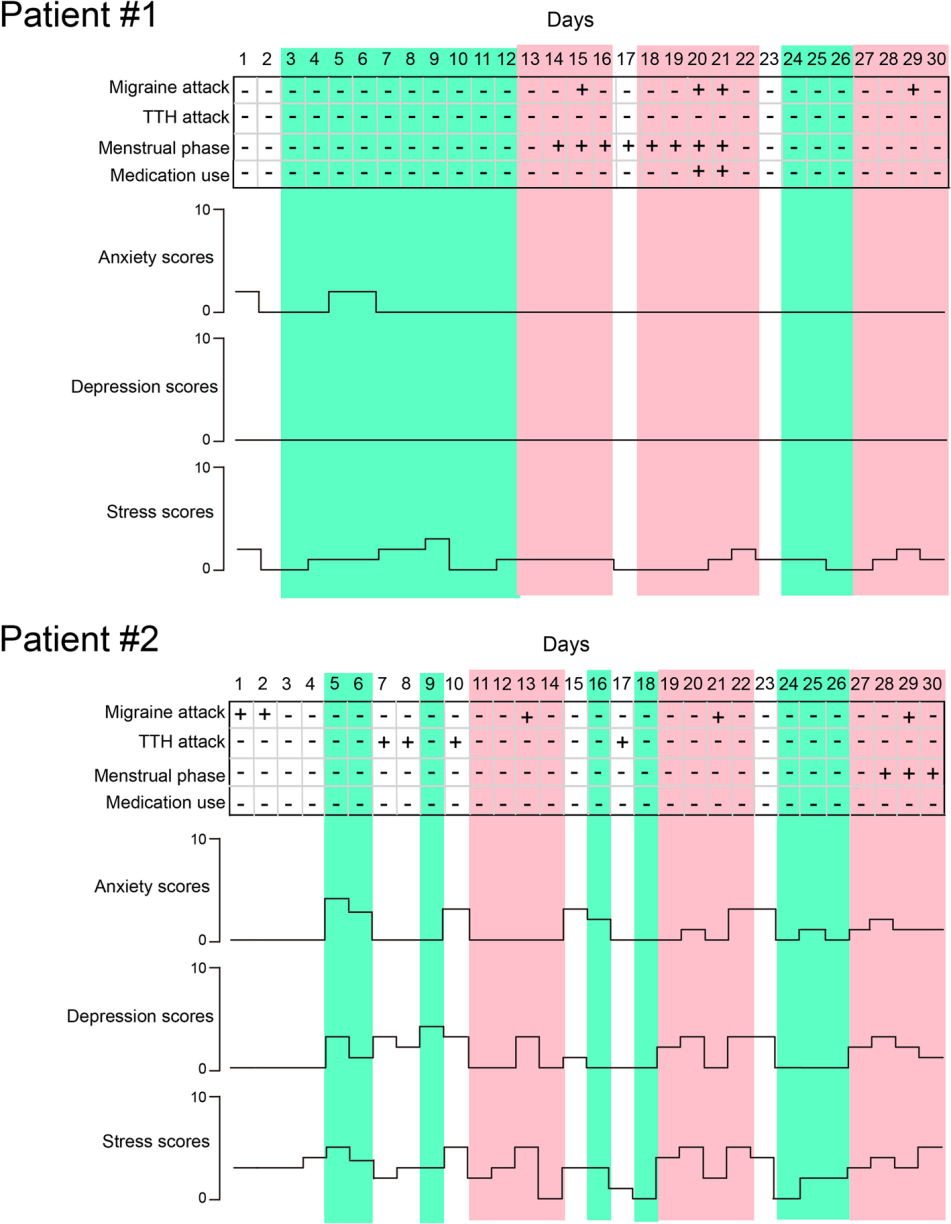
Headache attacks and clinical scores during 30-day period. Headache attack, menstrual phase, medication use, and psychometric scores within consecutive 30 days in two patients with EM. Days shaded in pink are parts of migraine cycles. Days shaded in aquamarine are in the pain-free interictal period. TTH, tension-type headache

The headache conditions and subjective psychometric scores of the two patients during the 30-day period are illustrated in Fig. 1. Patient 1 was a woman aged 32 years with a 20-year history of EM. During the 30-day recording period, three of her migraine cycles (shaded in pink in Fig. 1) qualified for analysis. In the preictal phase, she reported no aura but had prodromal symptoms of fatigue and yawning. Her migraine attacks were unilateral but not fixed to the right or left side. On days 20 and 21, 500 mg of acetaminophen were administered after EEG recording. Patient 2 was a 26-year-old woman with a 2-year history of EM. In the 30-day period, three of her migraine cycles (shaded in pink in Fig. 1) met the criteria. During the preictal phase, neither aura nor prodromal symptoms were reported. The patient’s headaches were unilateral but with no fixed side. Headache attacks on days 7, 8, 10, and 17 were considered TTHs because of their symptoms. For these two patients, a total of 21 days (shaded in green) were considered parts of interictal periods, but only 20 days of data were eligible for interictal analysis because a technical problem with the EEG device occurred on day 16 for patient 2. Both patients were free of cutaneous allodynia according to the baseline evaluation.

### Somatosensory-evoked responses during the migraine cycle

**Fig. 2:**
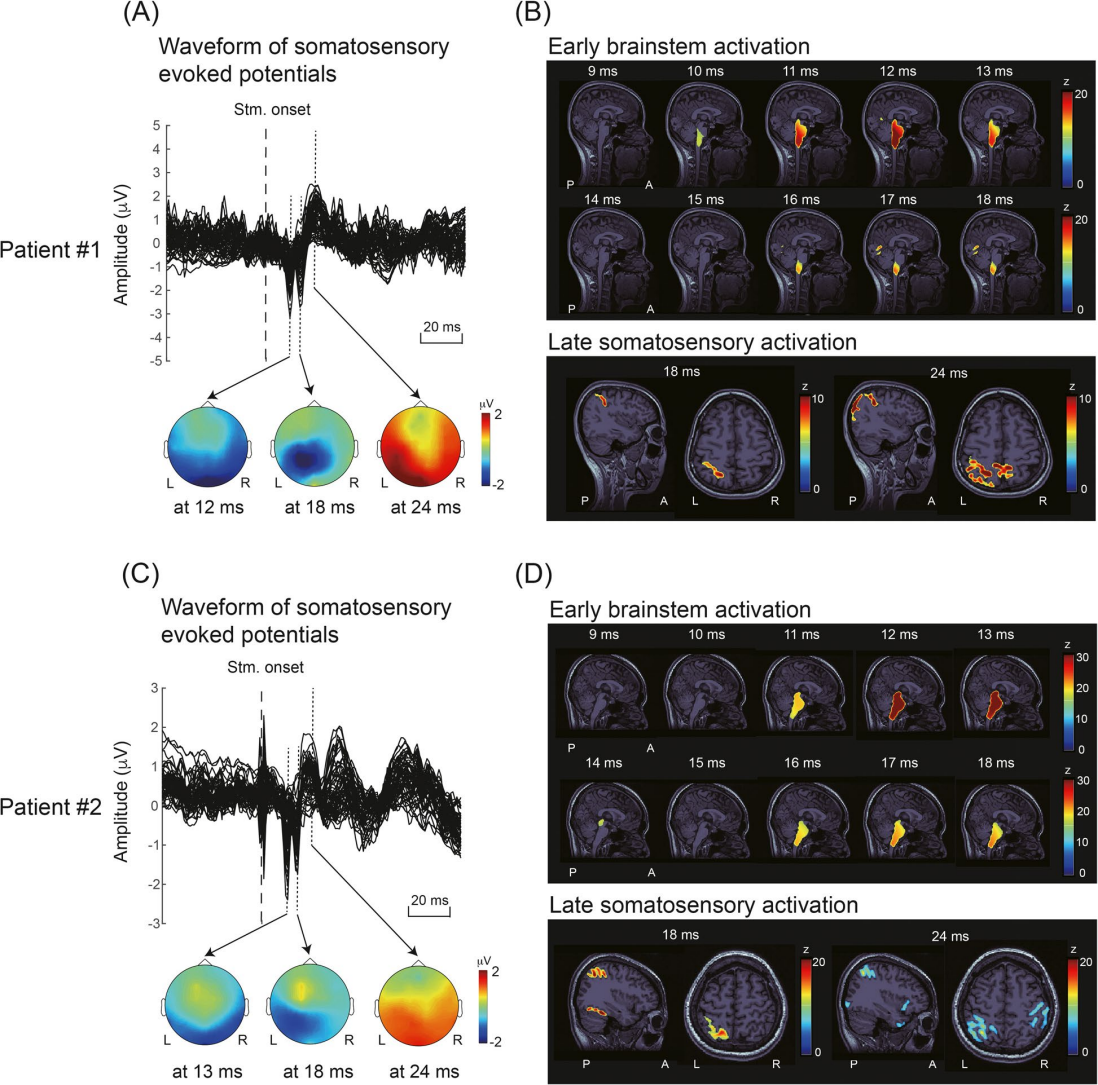
Representative somatosensory-evoked responses. **A** Superimposed SSEPs and corresponding topography at 12, 18, and 24 ms for patient 1. **B** Early (9–18 ms) and late (18 and 24 ms) activation for patient 1 mapped onto her MRI. **C** Superimposed SSEPs and corresponding topography at 13, 18, and 24 ms for patient 2. **D** Early (9–18 ms) and late (18 and 24 ms) activation for patient 2 mapped onto her MRI. Stim., stimulus; L, left; R, right; A, anterior; P, posterior

In response to somatosensory stimulation, the superimposed waveforms of evoked potentials from all electrodes are depicted in the upper panels of Fig. 2A and Fig. 2C for the preictal period for patient 1 (day 20) and patient 2 (day 12), respectively. Three prominent components peaked at 12 (or 13), 18, and 24 ms, and their corresponding topographies (lower panels of Fig. 2A and Fig. 2C) indicate subcortical and contralateral parietal activation. Figure 2B and D present the neural activation at 9–18, 18, and 24 ms mapped onto individual brain images. For both patients, the early component (12 or 13 ms) originated in the brainstem, and the later components (18 and 24 ms) originated in the S1.

**Fig. 3:**
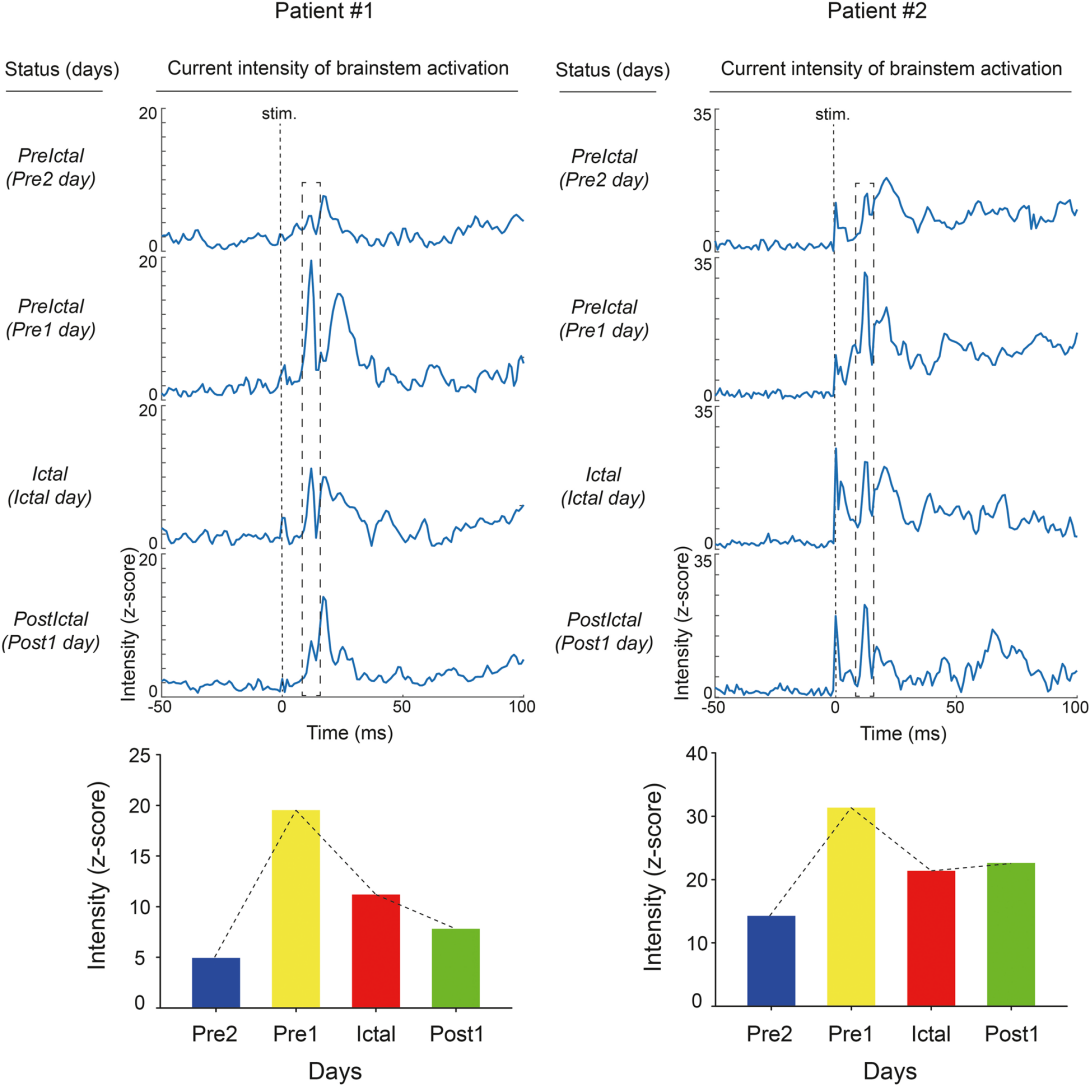
Evoked brainstem activation during migraine cycle. Top: Brainstem activation over time (− 50 to 100 ms) during a representative migraine cycle for patient 1 and patient 2. Bottom: Peak brainstem activation during migraine cycle. *Pre2*, 24–48 h before headache onset; *Pre1*, within 24 h before headache onset; *Ictal*, during migraine attack; *Post1*, within 24 h after headache offset. Stim., stimulus onset

**Fig. 4:**
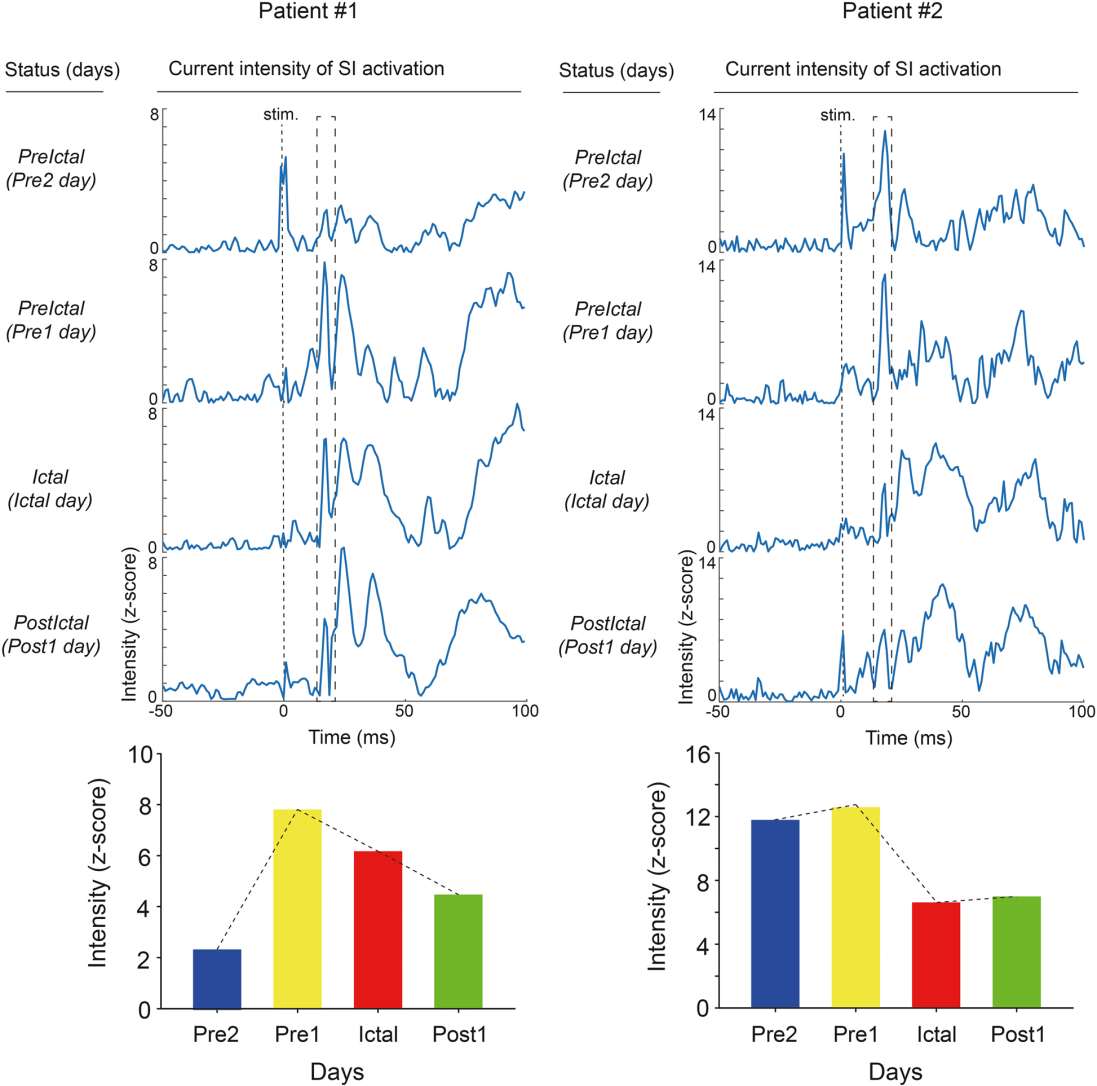
Evoked current intensity of S1 activation during migraine cycle. Top: S1 current intensity over time (− 50 to 100 ms) within one representative migraine cycle for patient 1 and patient 2. Bottom: Peak S1 activation during migraine cycle. S1, primary somatosensory cortex; *Pre2*, 24–48 h before headache onset; *Pre1*, within 24 h after headache onset; *Ictal*, during migraine attack; *Post1*, within 24 h after headache offset

The dynamic current intensity (*z* score) of brainstem activation for − 50 to 100 ms during one migraine cycle is presented in Fig. 3 for patient 1 (left) and patient 2 (right). The fluctuating brainstem activation was observed during the migraine cycle and the peak intensity of a clear component at 12–14 ms was obtained (lower part of Fig. 3), demonstrating the association between brainstem activation and the migraine cycle. Specifically, the largest brainstem activation in both patients was noted in the preictal phase on the *Pre1* day. Additionally, the S1 activation indicated dynamic current intensity during one migraine cycle in both patients (Fig. 4). The peak intensity of S1 activation was obtained at 18–19 ms, and S1 activation fluctuated within the migraine cycle. Similar to brainstem activation, S1 activation was the strongest in the preictal phase (*Pre1*).

**Fig. 5:**
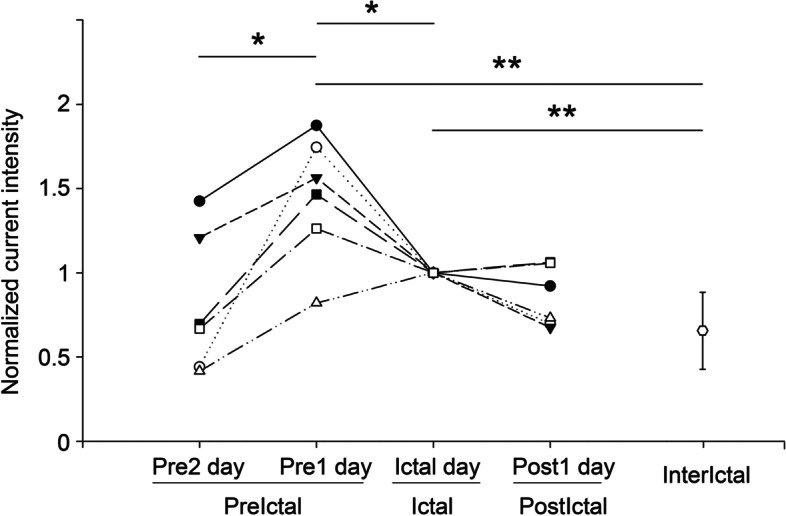
Brainstem activation during migraine cycle. Differences in normalised peak brainstem intensity among migraine cycles (*n* = 6) and migraine phases (*Pre2*, *Pre1*, *Ictal*, *Post1,* and *Interictal*). *, *P* < 0.05; **, *P* < 0.01; *Pre2*, 24–48 h before headache onset; *Pre1*, within 24 h before headache onset; *Ictal*, during migraine attack; *Post1*, within 24 h after headache offset; *Interictal,* the period ˃48 h between the last and next headache phase

**Fig. 6:**
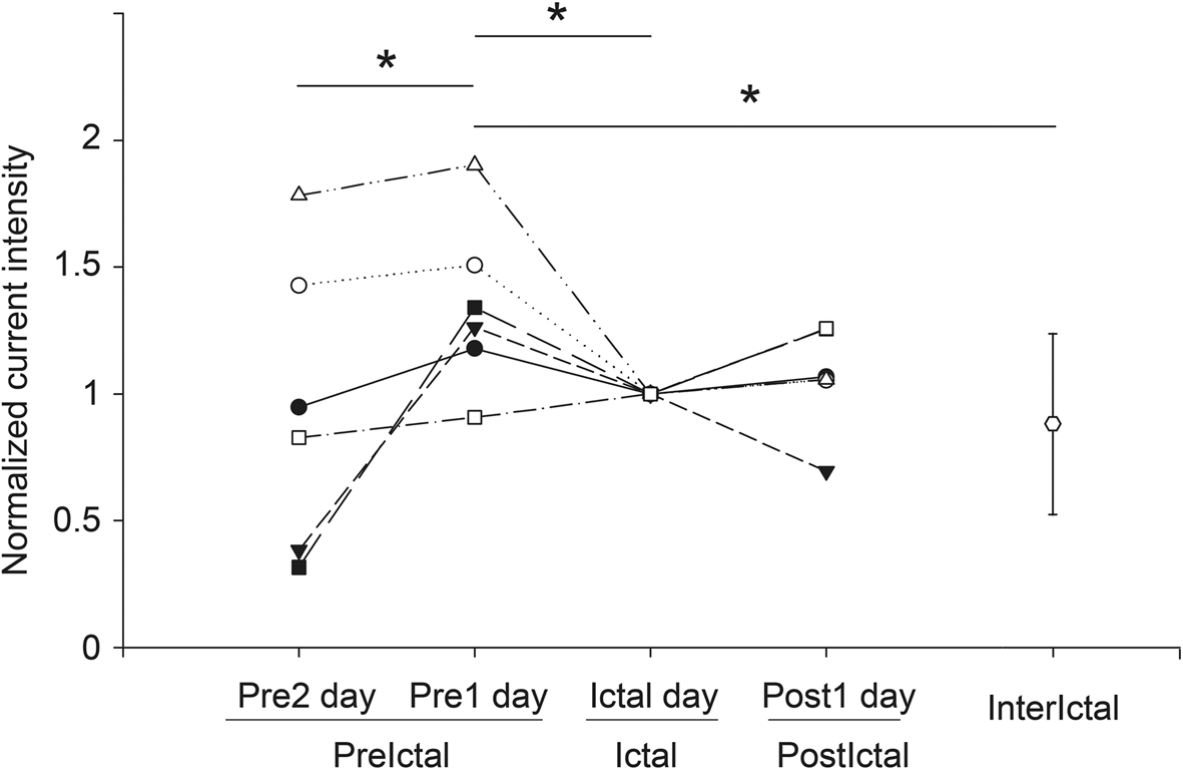
S1 activation during migraine cycle. Differences in normalised peak S1 intensity among migraine cycles (*n* = 6) and migraine phases (*Pre2*, *Pre1*, *Ictal*, *Post1,* and *Interictal*). *, *P* < 0.05; **, S1, primary somatosensory cortex; *Pre2*, 24–48 h before headache onset; *Pre1*, within 24 h before headache onset; *Ictal*, during migraine attack; *Post1*, within 24 h after headache offset; *Interictal,* the period ˃48 h between the last and next headache phase

The normalised current intensity in the brainstem was compared between phases of the same migraine cycle (*n* = 6 for each phase) and each day of the migraine cycle and interictal period (*n* = 20; Fig. 5). A significant difference in normalised current intensity was observed between phases (χ^2^ = 19.5, *P* = 0.001). The Wilcoxon signed-rank test indicated a significant increase in brainstem current intensity from *Pre2* to *Pre1* (*z* = 2.2, *P* = 0.028) and significant decrease from *Pre1* to *Ictal* (*z* = − 1.99, *P* = 0.046). Additionally, the normalised brainstem current intensity in the interictal period was lower than that in the *Pre1* (*z* = − 3.1, corrected *P* = 0.004) and *Ictal* (*z* = − 3.57, corrected *P* < 0.001) phases. In the comparison of the S1 activation on days within the migraine cycle and the interictal period (Fig. 6), significant differences in normalised current intensity were also observed between distinct days (χ^2^ = 13.1, *P* = 0.011). Specifically, a significant increase from *Pre2* to *Pre1* and a significant decrease from *Pre1* to *Ictal* were noted in the S1 intensity (both *P* < 0.05); moreover, the intensity in the interictal period was lower than it was in *Pre1* (*z* = − 2.58, corrected *P* = 0.04).

### Somatosensory gating profile during migraine cycle

**Fig. 7:**
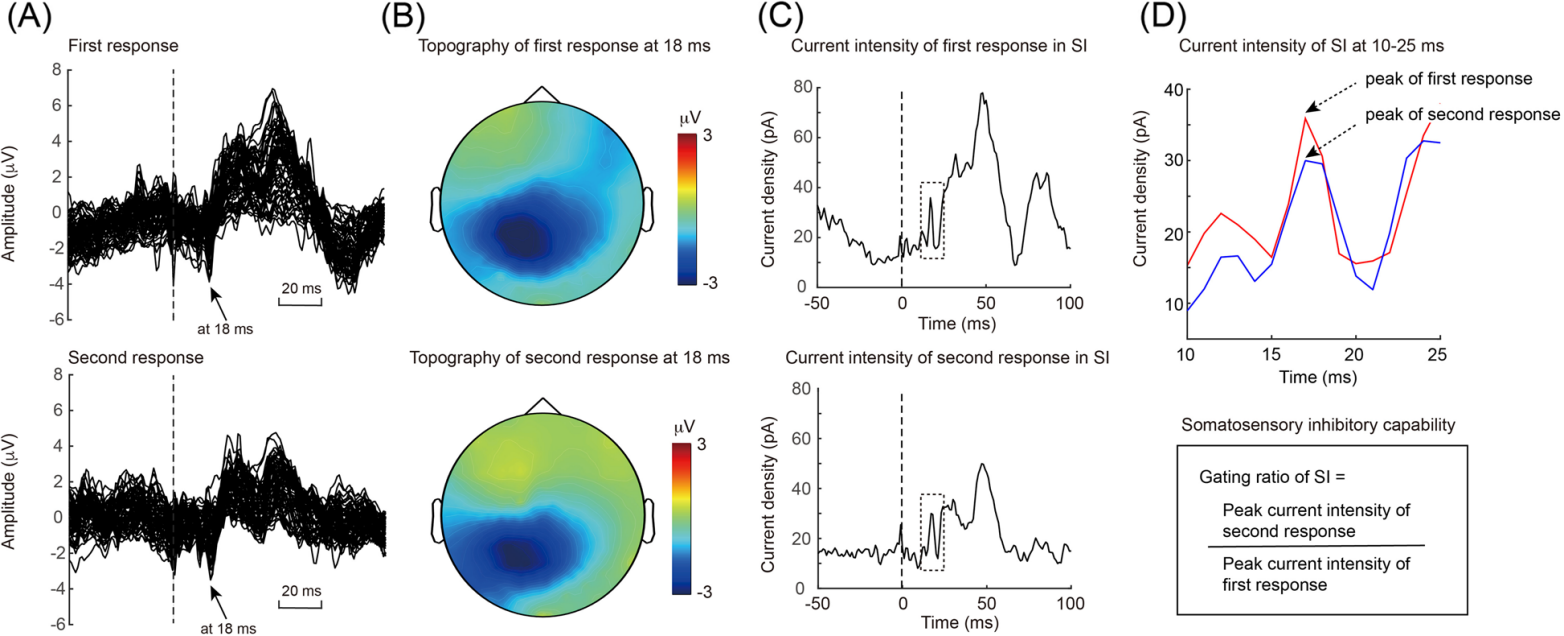
SSEPs for paired-pulse stimulation. **A** Superimposed SSEPs (first and second responses) peaked at 18 ms in response to paired electrical stimulation. **B** Topographies of peak first and second responses. **C** S1 current intensity over time for first and second responses. Dashed square indicates peak S1 component. **D** Acquisition and calculation of S1 gating ratio (inhibitory capability). S1, primary somatosensory cortex

**Fig. 8:**
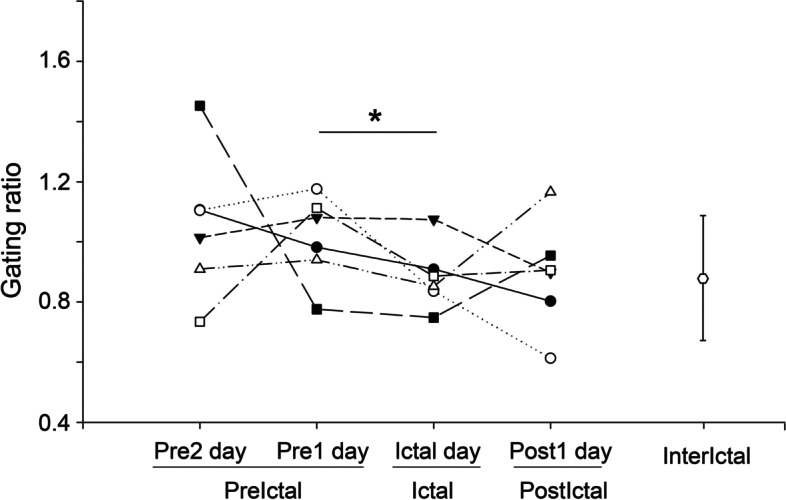
S1 inhibition during migraine cycle. Differences in S1 gating ratio among migraine cycles (*n* = 6) and migraine phases (*Pre2*, *Pre1*, *Ictal*, *Post1*, and *Interictal*). *, *P* < 0.05; S1, primary somatosensory cortex; *Pre2*, 24–48 h before headache onset; *Pre1*, within 24 h before headache onset; *Ictal*, during migraine attack; *Post1*, within 24 h after headache offset

The potentials evoked through paired-pulse stimulation are visualised in Fig. 7 for patient 1 during the interictal period. Prominent first and second somatosensory responses were elicited and peaked at 18 ms (Fig. 7A). The corresponding topography at 18 ms indicated contralateral somatosensory activation (Fig. 7B); moreover, the current intensity of the contralateral S1 peaked at 15–20 ms (Fig. 7C). The somatosensory inhibitory capability was determined from the gating ratio (Fig. 7D). The somatosensory gating ratios were compared across the migraine cycle (Fig. 8). The gating ratio was higher on *Pre1* days (1.01 ± 0.14) than on *Ictal* days (0.88 ± 0.11; *z* = 2.2, *P* = 0.028).

## Discussion

This study was the first to use consecutive daily EEG recordings for 30 days to elucidate the electrophysiological brainstem and S1 dynamics in terms of the neuronal excitability and inhibition during migraine cycles in patients with EM. The primary finding of this study is that the somatosensory excitability in both the brainstem (peaking at 12–14 ms after stimulus) and S1 (peaking at 18–19 ms after stimulus) reached its maxima on the day prior to the acute migraine attack (*Pre1*) and then decreased; the S1 inhibitory capability was lower in the *Pre1* than in the *Ictal* phase.

### Dynamic brain activation during the migraine cycle

During the migraine cycles, brainstem activation peaked level within 24 h before headache onset and subsequently declined during the migraine attacks. The brainstem activation in these two phases was also higher than it was during the interictal period. Neuroimaging findings have indicated the cyclic changes in brain activity during different phases of the migraine cycle, corroborating our observations of fluctuating activity [8, 11–13, 31–33]. Increased brainstem activation preceding the migraine attack has been observed after trigeminal nociceptive stimulation [33], after noxious orofacial stimulation [34], and in the resting state [31] and is consistent with enhanced resting-state functional connectivity among brainstem nuclei or between brainstem and cortical regions [13, 31]. In a diffusion tensor imaging study, mean brainstem diffusivity increased during the interictal period, but decreased prominently 24 h before an attack [35]. Moreover, greater brainstem activity during spontaneous or triggered migraine attacks than during interictal periods has been reported [36–39]. Using nonpainful somatosensory stimulation and EEG, this study verified the alterations in brainstem activation across the migraine cycle, which imply the dysfunctional brainstem functions immediately before and during an attack.

S1 excitability also fluctuated; in particular, S1 activation increased before attacks. This fluctuation in cortical excitability in migraine has been evidenced through amplitude measurements, power spectra, and habituation of contingent negative variation responses [40], resting-state EEG power and coherence [5], and beta event-related desynchronisation during sensorimotor tasks [41, 42]. Nonetheless, in MRI studies, functional connectivity and anisotropy of the thalamus also increases immediately before an attack [31, 35], but its interictal connectivity with the cortical networks [43] is disrupted during an attack [44]. These results suggest that the increased susceptibility to the neuropathological process before an attack might involve a facilitatory thalamocortical mechanism. Therefore, in patients with migraine, the somatosensory network may dynamically amplify afferent traffic or be cyclically hyperresponsive to peripheral stimuli that increase afferent traffic. Consistent with prior hypotheses [3, 38], dysregulation of the central excitability of the somatosensory system could play a primary role in migraine pathophysiology. Furthermore, the cyclic peak latency of evoked responses on *Pre1* days occurred earlier in the brainstem (12–14 ms) than it did in the S1 (18–19 ms), indicating that the major driver of migraine pathophysiology might originate in somatosensory neurotransmission in deep brain structures (possibly the brainstem), and S1 activation seems to be reciprocal to the cyclic changes in brainstem activation. In longitudinal 30-day fMRI studies, the relationship between brainstem and S1 activation during migraine phases has remained undetermined because of the trade-off between the spatial resolution and scanning regions in fMRI techniques.

### S1 inhibitory function during migraine cycle

We discovered that somatosensory gating capability (which is related to habituation) decreased before migraine attacks, a finding consistent with the identification of a preictal maximum habituation deficit [7], suggesting a pre-ictal increase of cortical excitability. The exact pathophysiological mechanism remains unclear, but the preictal dynamics of cortical sensorimotor inhibition have been associated with changes in cortical or thalamic interneuronal activity [41, 45]. The thalamocortical network is thus implicated in migraine pathophysiology [46], which may indicate that the cyclic S1 habituation and neural synchronisation of the somatosensory network are modulated to some degree by the thalamocortical neurotransmitter system. Moreover, consistent with previous findings [6], we observed no difference in S1 inhibitory function between the interictal and ictal phases in our patients with EM.

### Relevance for migraine pathophysiology

Following several years in which the brainstem was considered the ‘migraine generator’, attention has recently shifted to the ‘hypothalamus’. This is because sequential fMRI studies as a response to trigeminal nociceptive stimulation on the same patient have demonstrated that the hypothalamus is activated 24–48 h before headache onset, and increases its functional connectivity with the brainstem [11, 12]. Our results indicate that the S1 and brainstem both reach maximum activation 24 h before an attack. The timing of activation is similar to that revealed in the fMRI analysis. In previous neurophysiology studies, researchers have observed that the closer the onset of an attack is, the more the evoked activity of the parietal cortex decreases, eventually returning to normal during an attack [47]. Moreover, in patients with migraine, defects in descending modulatory circuits may contribute to the migraine attack [48]. No other study has verified S1 activity during the pre-attack period (i.e. 48 h before the onset of the pain phase). Overall, these data, together with the study results, suggest that somatosensory cortex activity plays a key role in the recurrence of migraine attacks.

### Limitations

This study has several limitations. First, limited by the spatial resolution of EEG, this study failed to elucidate the functional roles of specific nuclei, such as the trigeminal nuclei or dorsal pons, in the brainstem during the migraine cycle. Second, although in neuroimaging studies the hypothalamus activation is regarded as a neurological signature of migraine generation [11]; however, we did not observe hypothalamic activation in our EEG analysis. This could be attributed either to the insufficiency of the low signal-to-noise ratio for obtaining reliable hypothalamus activation data because of the limited trial number of SSEP responses, or simply because the innocuous somatosensory pathway does not have a hypothalamic relay station [24]. Third, the effects of psychological distress on the cyclic brainstem and S1 activation remain elusive. Fourth, one of our patients was diagnosed as having TTH; this might have confounded the findings. Although several evidences have indicated common cortical disinhibition between migraine and TTH [49] as well as a normal amplitude of visual evoked responses [50], brainstem auditory evoked potentials [51] and contingent negative variation [52] in patients with TTH, the effects of TTH on the cyclical brainstem and SI activation must be identified. Finally, our data were obtained from only two patients; further investigations using larger sample sizes are warranted.

## Conclusion

This study demonstrated that the migraineur’s brain is subject to cyclical changes in brainstem and S1 excitability, and the neural substrates involved in the underlying pathophysiological mechanism include both excitation and inhibition. In terms of future migraine treatment, stabilization of brain excitability may be the pivotal feature, and the key target for action may lie in deep brain structures (especially the brainstem). Further investigations with larger sample sizes are warranted.

## Data Availability

The datasets generated during and/or analyzed during the current study are available from the corresponding authors on reasonable request.

## Abbreviations

EM: Episodic migraine
S1: Primary somatosensory cortex
MNE: Minimum norm estimate
SSEPs: Somatosensory-evoked potentials
